# Exploring New Ways of Using Simulations for Teaching Accessibility with Gerontology and Urban Design Students

**DOI:** 10.1093/geroni/igaf122.4370

**Published:** 2025-12-31

**Authors:** Tamara Smith, Alina Gross

**Affiliations:** Westfield State University, Westfield, Massachusetts, United States; Westfield State University, Westfield, Massachusetts, United States

## Abstract

At Westfield State University two departments regularly teach undergraduate students about physical and cognitive accessibility in urban/interior design. One is the Health Sciences, offering courses such as GERO 101: Introduction to Gerontology and GERO 0102: Health and Physical Aging, and the other is Geography, Planning, and Sustainability, offering courses that cover these issues such as GPS 216: Social Justice and the City. Teaching undergraduate-level students about accessibility across a range of disabilities has a complicated history, particularly around the usage of so-called “simulations”, which ask students to do activities such as wear a blindfold to understand visual impairment, wear ear coverings to understand auditory impairment, or use a wheelchair to learn about physical disabilities. While there may be benefits to versions of these exercises and facilitators are likely well-meaning, disability simulations also risk of being offensive, oversimplified, insensitive, and ineffective in their purpose. As faculty from the above departments, our conference session will present ways that we have undertaken simulation-like activities while also addressing the concerns around such activities by reworking and reframing them in a variety of ways to address potential pitfalls. We will share how we have achieved some of the positive objectives of disability simulations, while also making sure students understand context and critiques of these activities appropriately. We will also share ways that we have students focus on the experience of these activities rather than emphasizing such activities will provide specific insights into a particular disability, and have students reflect on these activities in new ways.

